# High Upper Critical Field and Type II Superconductivity in Epitaxial Cubic W_2_N Thin Films

**DOI:** 10.1002/advs.77576

**Published:** 2026-09-06

**Authors:** Aditya Singh, Arnaud le Febvrier, Sanath Kumar Honnali, Abhisek Mishra, Grzegorz Greczynski, Subhankar Bedanta, Per Eklund, Ajay Soni

**Affiliations:** ^1^ School of Physical Sciences Indian Institute of Technology Mandi Mandi Himachal Pradesh India; ^2^ Department of Chemistry – Ångström Laboratory; Inorganic Chemistry Uppsala University Uppsala Sweden; ^3^ Laboratory for Nanomagnetism and Magnetic Materials (LNMM) School of Physical Sciences National Institute of Science Education and Research (NISER) An OCC of Homi Bhabha National Institute (HBNI) Jatni Odisha India; ^4^ Thin Film Physics Division Department of Physics, Chemistry, and Biology (IFM) Inköping University Linköping Sweden

**Keywords:** superconductivity, thin films, tungsten nitride, upper critical field

## Abstract

Transition Metal Nitrides (TMNs) are a versatile class of materials, combining chemical robustness, high hardness, and superconducting behaviour with critical temperatures between 2–10 K. While several binary TMNs have been explored, superconductivity in stoichiometric W_2_N has remained largely unexplored. Here, we report on superconducting thin films of stoichiometric W_2_N, demonstrating a distinctly high upper critical field (*H_c2_*) of ∼ 8.5 T, uncommon among binary TMNs. This robust superconducting response under high magnetic fields highlights the technological relevance of W_2_N for integrated quantum and cryogenic electronic platforms. Overall, these results position stoichiometric W_2_N as a promising addition to the TMN superconducting landscape, opening new avenues for functional materials design based on chemically stable and mechanically resilient nitrides.

## Graphical abstract

Superconducting thin films of stoichiometric W_2_N demonstrate a high upper critical field (*H_c2_*) of ∼ 8.5 T, uncommon among binary transition metal nitrides. This robust superconducting response under high magnetic fields highlights the technological relevance of W_2_N for superconducting applications, e.g., integrated quantum and cryogenic electronics.

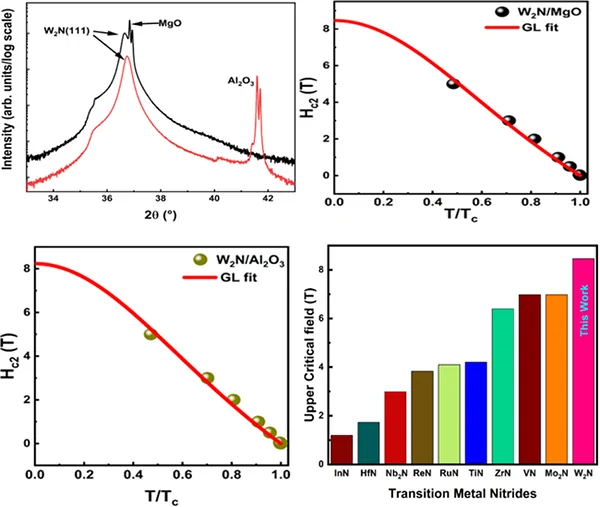

## 1 Introduction

Transition metal nitrides (TMNs) constitute a versatile class of materials characterized by a unique interplay of metallic and covalent bonding, which imparts an exceptional combination for mechanical robustness, thermal stability, and electronic tunability [1]. The bonding hybridization underlies their widespread use in applications such as diffusion barriers, protective coatings, and cutting tools [2, 3, 4, 5]. Beyond their mechanical resilience, TMNs exhibit rich electronic phenomena and applications, ranging from metal–insulator transitions [6], ferromagnetism [7], spintronic functionalities [8] and superconductivity [9]. The incorporation of nitrogen atoms strengthens the metal–nitrogen bonds and enhances electron–phonon (*e‐ph*) coupling, which plays a pivotal role in mediating superconductivity [10]. Several TMNs such as InN, HfN, ZrN, Mo_2_N, Nb_2_N, ReN, RuN, TiN, and VN [11, 12, 13, 14, 15, 16, 17, 18] have been explored for their low‐temperature electronic transport properties, underscoring their potential as tuneable superconducting platforms. Furthermore, TMNs can crystallize in several structural polymorphs, such as hexagonal [19], cubic [18], orthorhombic [20], rhombohedral [21], and tetragonal phases [22], each associated with distinct electronic structures and phonon dynamics. The superconducting properties are strongly structure‐dependent, for instance, hexagonal MoN exhibits a relatively high critical temperature (T_c_ ∼ 13 K) [23], whereas cubic Mo_2_N typically shows a lower T_c_ ∼ 5–6 K [18], revealing the sensitivity of superconductivity to lattice symmetry and bonding environment. Nitrogen stoichiometry also plays a decisive role, as even small deviations can significantly alter carrier concentrations, induce lattice strain, and modulate *e‐ph* coupling strength [24].

Among TMNs, tungsten nitrides (W–N) are particularly interesting due to their polymorphic nature and multiple applications such as mechanical hardness, diffusion barrier performance, catalytic activity, and electrochemical energy storage capabilities [25, 26, 27, 28, 29]. Multiple phases such as cubic W_3_N_4_, hexagonal and rhombohedral W_2_N_3_ [21], cubic WN [30], and cubic W_2_N, have been reported, each with distinct structural and electronic characteristics. However, despite this structural richness, the relationship between phase formation, nitrogen stoichiometry, and superconducting behaviour in the W–N system remains poorly understood. The physical properties of these materials are highly sensitive to the W:N ratio and crystallographic phase, thus controlling stoichiometry within a specific crystal structure is important for getting bespoke properties. Theoretical predictions [31] have suggested W_2_N as a potential superconductor coexisting with a possible charge‐density‐wave instability, yet experimental validation has been scarce. Prior studies have primarily focused on tungsten thin films synthesized under varying N_2_: Ar atmospheres, yielding multiphase materials comprising metallic W, nitrogen‐stabilized β‐W, and W_2_N inclusions [32, 33, 34]. Consequently, the intrinsic superconducting properties of stoichiometric W_2_N have remained ambiguous. Previous reports have indicated superconductivity near 1.3 K, only marginally higher than that of pure tungsten (T_c_ ∼ 0.011 K) [35], in sharp contrast to other TMNs where nitrogen incorporation substantially enhances *T_c_* for instance, in case of Nb, *T_c_* ∼ 9 K, while NbN has *T_c_* ∼ 18 K [36, 37]. Thus, in the case of W_2_N, the role of structure, stoichiometry, and bonding provides a platform to explore the superconductivity in this material.

In this work, we address this research gap by presenting the first comprehensive experimental investigation of superconductivity in stoichiometric W_2_N thin films. The films are grown via reactive DC sputtering on Al_2_O_3_ and MgO substrates with an average thickness of ∼ 270 nm, exhibiting a clear superconducting *T_c_* around ∼ 5*K*. The superconducting behaviour is further corroborated by a distinct diamagnetic transition observed within the same temperature range, confirming the bulk nature of the superconductivity. Magnetization and resistivity measurements at different applied magnetic fields are employed to determine the lower (*H_c1_*) and upper critical fields (*H_c2_*), revealing values in the range of *H_c1_* ∼50 Oe and *H_c2_* ∼8.5 T, respectively. This comparatively high upper critical field, coupled with a relatively large intermediate state, indicates that W_2_N shows strong type‐II superconductivity. This characteristic is particularly significant for potential technological applications, such as in the development of high‐performance single‐photon detectors and other superconducting devices.

## 2 Results and Discussion

The optimized growth parameters were determined through preliminary depositions performed on c‐sapphire where the duration was limited to 30 mins. During that process systematic XRD analyses and elemental composition analysis by XPS were performed. While typically a rapid, surface‐sensitive tool for synthesis optimization, XPS was used here to determine the metal‐to‐nitrogen ratio and the thickness for optimizing thin film growth and to get the desired stoichiometry before depositing thicker films. In the optimized conditions described in the present article, the composition extracted from the XPS analysis shows a near‐stoichiometric composition with an N/W ratio of 0.52 (W_2_N_1.04_) (Table 1). A thickness of 54 nm was extracted from XRR analysis yielding a deposition rate of 1.8 nm/min (Figure S1). In the purpose of measuring transport properties, thicker films of 270 nm were deposited under the same condition on the same day and on two different substrate (c‐sapphire and MgO (111)). The 54 nm and 270 nm thick film on c‐sapphire have the same XRD pattern highlighting the reproducibility in short time span of the deposition process (Figure S2).

**TABLE 1:** Composition analysis using ERDA, RBS and XPS.

Sample	XPS	ERDA and RBS (at. %)				Chemical formula
	N/W	N/W	W	N	O	
270 nm W_2_N/MgO	—	0.57	63.8	36.2	—	W_2_N_1.14_
270 nm W_2_N/Al_2_O_3_	—	0.54	63.8	34.3	1.8	W_2_N_1.08_
54 nm W_2_N/Al_2_O_3_	0.52	—	—	—	—	W_2_N_1.04_

To achieve a more reliable and bulk‐sensitive compositional analysis of the thick films deposited on c‐sapphire and MgO (111), a combination of RBS and ToF‐ERDA was utilized. This dual approach is necessary to accurately quantify the heavy element (tungsten) and the lighter element (nitrogen), overcoming their strong mass contrast. Table 1 summarizes these bulk results alongside the XPS surface compositions obtained during the optimization process. The RBS and ToF‐ERDA analyses confirmed a composition close to the ideal 2:1 tungsten‐to‐nitrogen ratio. Specifically, W_2_N/Al_2_O_3_ exhibits a composition of W_2_N_1.08_, whereas W_2_N/MgO gives W_2_N_1.14_. These minor deviations from exact stoichiometry fall well within the typical tolerance range for sputtered transition‐metal nitrides and are consistent with stable W_2_N phase formation. Additionally, a minor oxygen signal (∼1.8 at. %) was detected in the W_2_N/Al_2_O_3_ film. Overall, these findings provide strong evidence for the successful deposition of near‐stoichiometric W_2_N thin films on both substrates.

**FIGURE 1:**
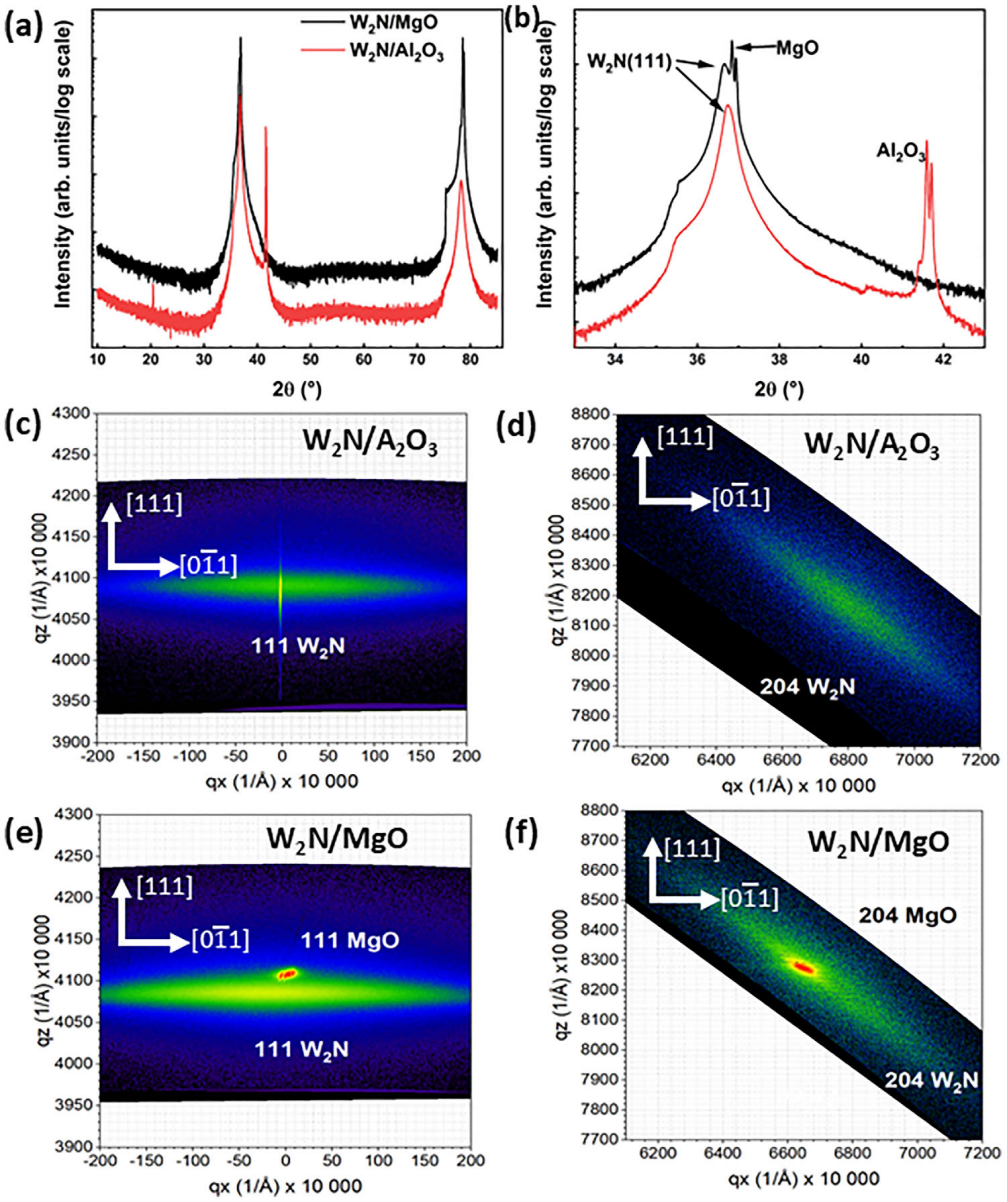
XRD patterns of W_2_N/MgO and W_2_N/Al_2_O_3_ in (a)10°–85° and (b) 33°–43°. RSM of (c,d) W_2_N/Al_2_O_3_ and (e,f) W_2_N/MgO films around the symmetric 111 and asymmetric 204 reflections, respectively. Note that qx and qz are expressed in units of 10^4^ (Å^−^^1^) for convenient representation.

Figure 1a shows the full‐range XRD 2*θ* scan for both W_2_N/Al_2_O_3_ and W_2_N/MgO, confirming the overall phase purity of the films without any detectable secondary phases across the scanned range. Figure 1b presents the magnified 2*θ* window around 34–42°, where the dominant film and substrate peaks appear more clearly. The substrate reflections are identified at 2*θ* ∼ 41.6° and at 2*θ* ∼ 36.8° identified at 0001 Al_2_O_3_ (PDF 00‐046‐1212) and 111 MgO (PDF 01‐071‐6452). The diffraction peak observed at 2*θ* ∼ 36.7° (d‐spacing = 2.447 Å) for W_2_N/Al_2_O_3_ and at 2*θ* ∼ 36.6° (d‐spacing = 2.453 Å) for W_2_N/MgO is indexed to the 111 reflection of cubic W_2_N (PDF 00‐025‐1257), indicating a preferential out‐of‐plane (111) orientation. Pole Figure analysis (Figure S3) has been used to determine the epitaxial relationship for both films, which are as follows: (111) W_2_N || (0001) Al_2_O_3_ (out‐of‐plane) and [11$\bar{2}$]W_2_N || [10$\bar{1}$0] Al_2_O_3_ (in‐plane); and (111) W_2_N || (111) MgO (out‐of‐plane) and [110] W_2_N || [110] MgO (in plane).

To further analyse the epitaxy, lattice coherence with the substrate, and strain of the films, Reciprocal Space Mapping (RSM) has been performed for both films. Figure 1c–f display the symmetrical RSM around the symmetric 111 reflections and the asymmetric 204 reflection measured on both films. Note that the out‐of‐plane direction (*q_z_*) corresponds to the [111] direction, while the in‐plane direction (*q_x_*) corresponds to the [$0\bar{1}1$] in‐plane of W_2_N. The substrate peaks are not visible and not shown in the RSM window for W_2_N/Al_2_O_3_ due to the different crystal symmetries between substrate and film (rhombohedral Al_2_O_3_ and cubic W_2_N), but all measurements have been performed after alignment on the substrate peak at the symmetrical position. The symmetrical RSM confirms the Bragg Brentano measurement, while the asymmetrical RSM confirms the epitaxial character of the films, with the possibility to resolve the structure of W_2_N. On c‐plane sapphire (Figure 1c,d) the extracted lattice constants (*a* = 4.188 Å, α  =  90.62°) reveal an in‐plane strain of +0.52% and out‐of‐plane strain of –1.09%. On MgO (111) (Figure 1e,f), reflections from both the substrate and film overlap but, similarly to c‐sapphire, indicate a small deviation from a cubic symmetry. The extracted lattice parameters (*a* = 4.190 Å, α  =  90.67°) reveal that the film has an in‐plane strain of +0.58% and an out‐of‐plane strain of –1.19%. On both substrates, W_2_N prosses relatively the same structure and lattice parameters with a slight deviation from a cubic cell with a cell parameter close to *a* ≈ 4.19 Å, α  =  90.6 – 90.7 °. The rocking curve FWHMs of the 111 peaks have been extracted from the symmetrical RSM and are 3.1° and 3.0° for the film deposited on sapphire and MgO respectively. These values reflect the mosaic spread (orientational dispersion) of the crystallites, although such broadening may generally arise from factors such as strain gradients, defects, twin domains, or compositional inhomogeneity. Comprehensive structural, compositional, and crystallographic characterization confirms that the synthesized thin films are single phase W_2_N, like NaCl structure, with half‐filled nitrogen sites (N/W ratio = 0.55 ± 0.02), providing a solid foundation for the investigation of their low‐temperature transport properties.

**FIGURE 2:**
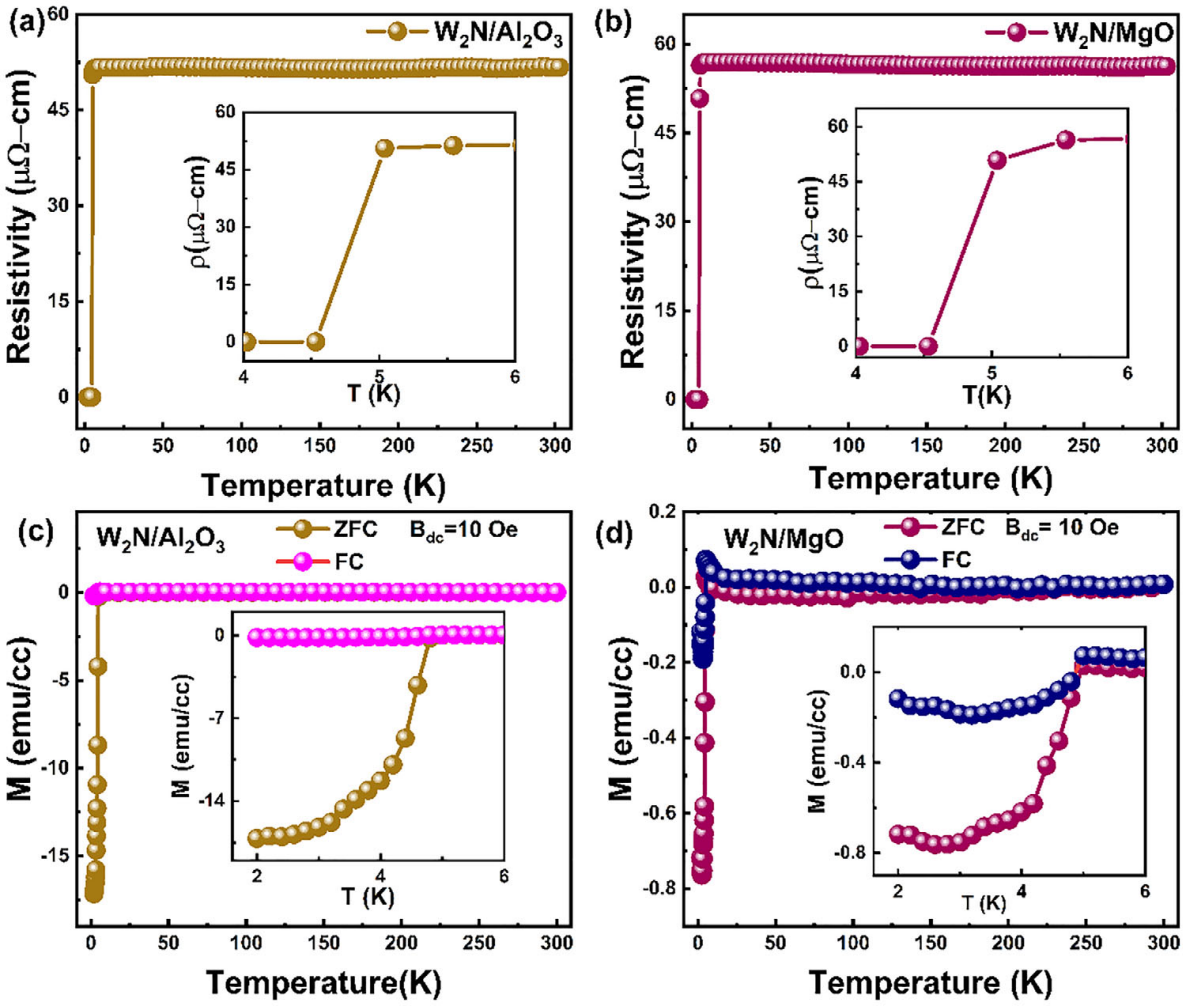
Temperature dependent Resistivity (*ρ(T)*) of (a) W_2_N/Al_2_O_3_​ and (b) W_2_N/MgO, ZFC and FC *M*‐*T* curves for (c) W_2_N/Al_2_O_3_​ and (d) W_2_N/MgO, (insets: low‐temperature data).

Figure 2a,b shows the temperature‐dependent resistivity plots of the thin films. The critical temperature (*T_c_*) for both films has been evaluated using the 90% criteria ($T_{c}^{onset}$), defined as the temperature at which the electrical resistivity drops to 90% of its normal‐state value. Using this criterion the *T_c_* is found out to be ∼ 5 *K* (for W_2_N/Al_2_O_3_) and ∼ 5.06 *K* for (W_2_N/MgO) with a transition width (Δ*T_c_*)  =  1 *K*. Further onset of superconductivity is corroborated by the DC magnetization measurements (Figure 2c,d) with both films showing a sharp diamagnetic transition around ∼ 5 K. The small residual strains in the films (∼0.5% in‐plane) are not expected to significantly influence *T_c_*, consistent with the nearly identical values observed on both the thin films.

**FIGURE 3:**
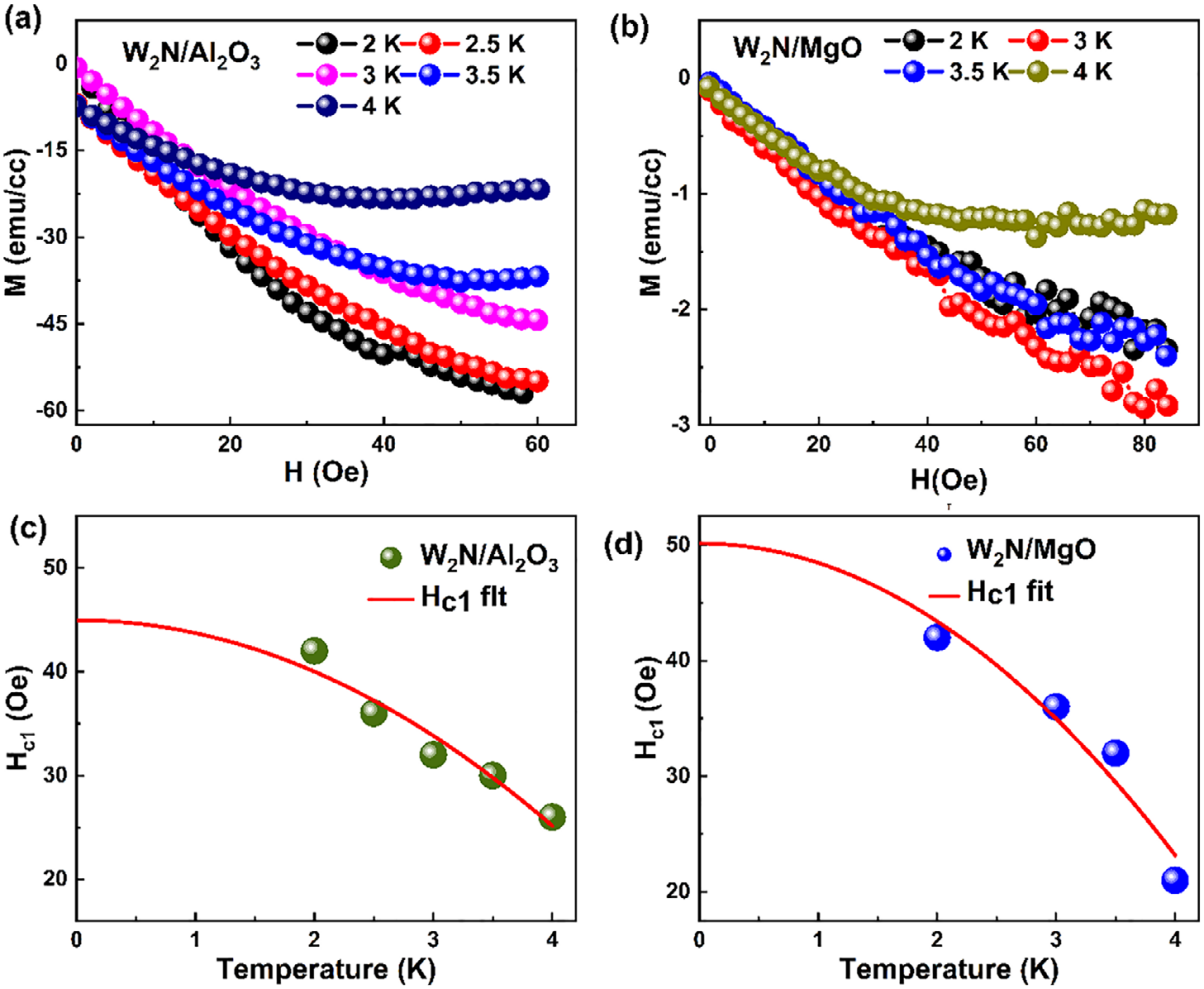
M‐ H plots (a,b) and H_c1_ versus T (c,d) plots of W_2_N/Al_2_O_3_ and W_2_N/MgO, respectively. The red line denotes the fitting using the model provided in the text.

The lower critical field (*H*_*c*1_ *(T)*) at different temperatures, have been estimated from the magnetic hysteresis (*M*‐*H)* measurements performed below T_c_ (Figure 3a,b) by using the Meissner–Ochsenfeld criterion [18]. Furthermore, to extract the *H*_*c*1_(0) the *H_c1_* vs *T* data has been fitted using the equation $\;H_{c1}=H_{c1}\;\left(0\right)\left[1-{\left(\frac{T}{T_{c}}\right)}^{2}\right]$ as shown in Figure 3c,d. The obtained values of *H*_*c*1_(0) are found to be ∼ 45 Oe for W_2_N/Al_2_O_3_ and ∼ 50 Oe for W_2_N/MgO.

**FIGURE 4:**
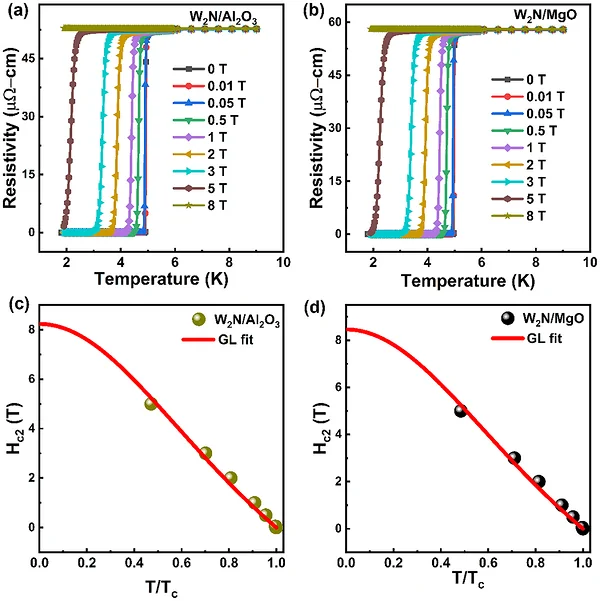
Low temperature resistivity at different applied magnetic field for (a)W_2_N/Al_2_O_3_, (b) W_2_N/MgO. *H_c2_* vs *T/T_c_* for (c) W_2_N/Al_2_O_3_, (d) W_2_N/MgO. The red line denotes the fitting of *H_c2_* with GL model [18].

The *H_c2_* is a crucial metric determining the operational range of superconductors in high‐field applications. Low temperature magnetoresistivity data (Figure 4a,b) reveal the shifting of critical temperature to the lower side, a characteristic feature of type II superconductors. *H_c2_* has been estimated from the $T_{c}^{onset}$ criteria, where the normal state resistivity value is taken at 6 K. The obtained *H_c2_* vs *T* have been fitted using the Ginzburg Landau (GL) relation to obtain *H*_*c*2_(0), given by $\;H_{c2}\;\left(T\right)=H_{c2}\;\left(0\right)\frac{\left(1-t^{2}\right)}{\left(1+t^{2}\right)},$ where *t* is the reduced temperature given by $\;t=\frac{T}{T_{c}}\;$ and *H*_*c*2_(0) is critical field at 0 K. *H*_*c*2_(0) estimated from GL model is found to be ∼ 8.23 T and ∼ 8.46 T for W_2_N/Al_2_O_3_ and W_2_N/MgO, respectively, as shown in Figure 4c,d. The superconducting performance of the W_2_N thin films is significantly enhanced compared with previously reported bulk WN, with (T_c_ ∼ 5) K and (H_c2_ ∼ 8.2) T, compared with (T_c_ = 2.86) K and (H_c2_ = 1.96) T for bulk WN [38]. Notably, the observed (H_c2_) is more than three times higher than that reported for bulk WN, highlighting the enhanced superconducting robustness of the W_2_N thin‐film phase. The estimated *H_c2_* values for W_2_N rank among the highest reported for transition metal nitride superconductors [11, 12, 13, 14, 15, 16, 17, 18], highlighting the high‐field robustness of these epitaxial films. The only class of transition metal nitrides with consistently higher upper critical fields are the conventional NbN‐based superconductors [39, 40, 41].

The *H_c2_(0)* is related to the normal state resistivity (ρ_*n*_) by the relation given by Werthamer–Helfand–Hohenberg (WHH) approximation given by $H_{c2}\;\left(0\right)=0.69\;T_{c}\frac{4eck_{b}}{\pi }N\left(0\right)\rho_{n}$, where *e*, *c*, *k_b_*, *N*(0) corresponds to electronic charge, speed of light, Boltzmann constant, density of states at Fermi level respectively [17]. The high *H_c2_(0)* in W_2_N can be ascribed to a combination of factors such as high *T_c_*, higher *N(0)* and high ρ_*n*_ as compared to the similar family of compounds [11, 12, 13, 14, 15, 16, 17, 18]. Further, we have extracted the zero‐temperature orbital limited upper critical field $H_{c2}^{orb}\left(0\right)$ from the WHH formula given as $\;H_{c2}^{\mathrm{orb}}\left(0\right)=-0.693T_{c}{\left(\frac{dH_{c2}}{\mathrm{dT}}\right)}_{T=T_{c}\;}$ [18]. The calculated value of *H*_*c*2_(0) is 6.67 *T* for W_2_N/Al_2_O_3_ and 6.82 *T* for MgO‐111. The obtained *H*_*c*2_(0) from WHH approximation is lower than the ones obtained from GL fitting. Both these critical fields are less than Pauli Paramagnetic limit which is given by *H_p_* =  1.86*T_c_* (9.3 T for W_2_N/Al_2_O_3_ and 9.41 T for W_2_N/MgO and therefore indicating of a conventional BCS type superconductivity. In order to gain a deeper insight about the mechanisms governing the upper critical field, we have calculated the Maki parameter (α), which is given by $\alpha =\sqrt{2}\;\frac{H_{c2}^{orb}\left(0\right)}{H_{p}\left(0\right)}$ [18]. In general, superconductivity can be destroyed either by the orbital pair‐breaking mechanism, where the Lorentz force on Cooper pairs suppresses phase coherence, or by the Pauli paramagnetic effect, where Zeeman splitting of electronic spins breaks the singlet pairing. The competition between these two mechanisms determines the limiting behaviour of *H_c2_*, with the Pauli limit becoming increasingly important in clean superconductors with short coherence lengths and large g‐factors. The obtained values of α are ∼1.01, 1.02 for W_2_N/Al_2_O_3_ and W_2_N/MgO, respectively. The observed α exceeding 1 signifies that the Pauli paramagnetic limiting effect is substantial and comparable to, orbital pair‐breaking [42]. Although α < 1.8, still greater than 1, thereby suggesting a potential formation of a Fulde‐Ferrell‐Larkin‐Ovchinnikov (FFLO) superconducting state, where Cooper pairs exhibit a finite momentum due to the spin imbalance induced by the magnetic field [43]. The GL coherence length (ξ_*GL*_), which characterizes the spatial scale over which the superconducting order parameter, recovers in the presence of perturbations, given by $\xi_{GL}=\sqrt{\frac{\phi_{0}}{2\pi H_{c2}\left(0\right)}}\;$, where $\phi_{0}=2.068\times 10^{-15}\;T.m^{2}$ is magnetic flux quantum, as calculated for both the films and found out to be ∼ 6.32 nm for W_2_N/Al_2_O_3_ and ∼ 6.23 nm for W_2_N/MgO. The GL London penetration depth (λ_*GL*_), which measures penetration of magnetic flux into a superconductor before being screened by supercurrents, is evaluated from $H_{c1}=\frac{\phi_{0}}{4\pi \lambda_{GL}^{2}}\;\ln\left(\frac{\lambda_{GL}}{\xi_{GL}}\right)$, and is found to be ∼ 388 nm for W_2_N/Al_2_O_3_ and ∼ 366 nm for W_2_N/MgO. Furthermore, we have also evaluated GL parameter (κ_*GL*_) given by $\kappa_{GL}=\frac{\lambda_{GL}}{\xi_{GL}}\;$, a strong indicator of type of superconductivity presents in the system and found to be ∼56 for W_2_N/Al_2_O_3_ and ∼59 for W_2_N/MgO, significantly larger than $\frac{1}{\sqrt{2}}$, indicating a strong type‐II superconductivity in both the synthesized thin films. The thermodynamical critical field (*H_c_*) is evaluated using the relation *H*_*c*1_(0).*H*_*c*2_ (0) = *H_c_* ^2^ ln κ_*GL*_, which is found to be ∼ 960 *Oe* and ∼ 1020 *Oe* for W_2_N/Al_2_O_3_ and W_2_N/MgO respectively. All the estimated parameters for thin films are tabulated in Table 2.

**TABLE 2:** Thin Film characteristics of W_2_N on different substrates.

Sample	t (nm)	*T_c_* (*K*)	*H_c1_(0)* (*Oe*)	*H_c2_(0)* (*T*)	$H_{c2}^{orb}\left(0\right)$ (*T*)	H_c_ (*Oe*)	ξ_*GL*_ (nm)	λ (nm)	α
W_2_N/Al_2_O_3_	270	5	45	8.23	6.67	960	6.32	388	1.01
W_2_N/MgO	270	5.06	50	8.46	6.82	1020	6.23	366	1.02

## 3 Conclusion

In summary, we demonstrate that stoichiometric W_2_N thin films exhibit robust type‐II superconductivity with an upper critical field of ∼ 8.5 *T*, positioning them among the strongest magnetic field resilient binary transition metal nitrides known to date. Analysis of the Maki parameter (α ∼ 1) indicates that Pauli paramagnetic effects are comparable to orbital pair‐breaking. This theoretical analysis suggests that W_2_N could serve as a promising platform for exploring exotic superconducting states such as the FFLO phase. The observed type‐II superconductivity highlights W_2_N as a possible material for both fundamental quantum materials research studies and the development of next‐generation superconducting technologies.

## 4 Experimental Section

W_2_N thin films were grown on *c*‐plane sapphire (W_2_N/Al_2_O_3_) and MgO (111) (W_2_N/MgO) substrates by reactive DC magnetron sputtering in an ultrahigh vacuum chamber [44]. Prior to deposition, both substrates were cleaned sequentially with a Hellmanex soap solution (3 min), deionized (DI) water (5 min × 2), and then sonicated in acetone (10 min) and ethanol (10 min) and were annealed at ∼ 800°C (in the deposition chamber) for 2 h. These wet cleaning steps are particularly important for MgO substrate to help remove surface hydroxides and carbonates [45]. The optimum deposition condition for obtaining W_2_N were a substrate temperature of ∼ 200°C, a deposition pressure of ∼ 0.32 Pa with a mixture of N_2_/Ar with a flow ratio of ∼ 41 sccm/ 18 sccm 70% N_2_ in the plasma, a target power of 44 W. The base pressure of the deposition chamber at deposition temperature was 2.4 × 10^−9^ Torr. Before depositing the final thick W_2_N film, several preliminary depositions were carried out on which structure and composition were analysed systematically. X‐Ray Photoelectron Spectroscopy (XPS) and X‐Ray Reflectivity (XRR) were performed on a thinner W_2_N film grown under identical deposition conditions as the samples presented in this manuscript to determine stoichiometry and deposition rate.

XPS analyses were performed during the deposition conduction optimization on film of roughly 50 nm. The sample was transferred from the UHV deposition chamber to the XPS system with less than 3 min of atmospheric exposure to minimize contamination. XPS analyzes of W_2_N films were conducted in an Axis Ultra DLD instrument from Kratos Analytical (UK) with the base pressure lower than 1.1 × 10^−9^ Torr (1.5 × 10^−7^ Pa). Monochromatic Al Kα radiation (hν = 1486.6 eV) was used with the anode power set to ∼ 150 W, and the core level spectra W (*4f*) and N (*1s*) were recorded at normal emission angle. The analyzer pass energy was set at 20 eV, yielding a full width at half maximum of Ag 3d_5/2_ peak (∼ 0.55 eV) of Ag foil as a reference. To ensure accurate N/W quantification, samples were analyzed in the as received state, thus avoiding preferential N re‐sputtering by Ar^+^ ions [46]. The analysis area was 0.3 × 0.7 mm^2^. Spectrometer calibration was verified by measuring Au 4f_7/2_, Ag 3d_5/2_, and Cu 2p_3/2_ peak positions from sputter‐etched Au, Ag, and Cu samples and comparison to the recommended ISO standards for monochromatic Al Kα sources [47]. Analyses of XPS data were done using CASA XPS software for a simple quantification of the W/N ratio by applying, on the high resolution core level spectra, a Shirley background to retrieve the peak intensity and using their respective relative sensitivity factors.

Further elemental composition analysis on the thicker film was measured by a combination of time‐of‐flight‐Elastic Recoil Detection analysis (ToF‐ERDA) and Rutherford Backscattering Spectrometry (RBS). The measurements were carried out using the Pelletron Tandem accelerator (5MV NEC‐5SDH‐2) [48]. The ToF‐ERDA measurements were conducted using 36 MeV primary iodine ion (^127^I^8+^) beam. The incident beam angle to the target surface normal was 67.5°, while the ToF‐telescope and the gas ionization detector were placed at 45° relative to the incident beam direction. The depth profile of the elemental composition was acquired from the ToF‐ERDA time and energy coincidence spectra using the Potku (version 2.2.4) code [49]. For RBS measurements, a 2 MeV He^+^ primary beam was used, incident at 5° to the sample surface normal and the backscattered ions were collected at 170° and the recorded spectra were analysed using SIMNRA (version 7.03) software [50]. The RBS measurements were performed after T ToF‐ERDA.

X‐ray diffraction (XRD) measurements were performed on a PANalytical X'Pert PRO diffractometer in Bragg–Brentano (θ–2θ) geometry with a Cu Kα (λ = 1.540598 Å) radiation and Ni filter. The recorded 2θ range is 10–90^0^ with a step size of 0.008^0^ and an equivalent time/step of 19 sec using the PIXcel 1D detector. Pole Figures were acquired with a polycapillary with a crossed slits (4 mm^2^) as primary optics and parallel plate collimator (0.18°) (phi steps size = 1° and chi step size = 1°). Reciprocal Space Maps (RSM) of the symmetric 111 peak and asymmetric 204 peak of W_2_N were acquired on the same type of diffractometer equipped with a hybrid mirror as primary optics and a PiXcel2D detector for fast acquisition with no optics for the receiving optics. The XRR was performed using x‐ray mirror with 1/32° divergence slit in primary and parallel plate collimator in secondary optics. All RSM measurements were performed after proper ω, 2θ, ψ alignment on the substrate. Symmetric and asymmetric reflections were used to determine the lattice geometry and extract the lattice parameters assuming a cubic W_2_N structure oriented along the (111) direction (*a*  =  *b*  =  *c*). The experimentally determined in‐plane and out‐of‐plane *d*‐spacings were then compared with the corresponding unstrained cubic reference values to evaluate the lattice distortion and strain state of the films.

The low temperature electrical transport (2‐300 K) measurements were carried out using the physical property measurement system (PPMS, Quantum Design) equipped with a 14 T magnet. The temperature dependent magnetization measurements were carried out using an MPMS3 SQUID‐VSM magnetometer (Quantum Design make) in Zero‐Field‐Cooled (ZFC) and Field‐Cooled (FC) protocols. The samples were first cooled to the base temperature (2K) at zero magnetic field, after which 10 Oe field was applied and the magnetization was recorded. Subsequently, the sample was cooled in the applied field, and the magnetization was measured again during the warming cycle under the same field conditions.

## Author Contributions

**Aditya Singh**: conceptualization, investigation, writing – original draft, methodology, validation, formal analysis, data curation. **Arnaud le Febvrier**: investigation, writing – review and editing, funding acquisition, validation, formal analysis, methodology. **Sanath Kumar Honnali**: investigation, writing – review and editing, methodology, formal analysis, validation, data curation. **Abhishek** and **Subhankar**: data curations, methodology, formal anlaysis, validation and writing – review editing. **Grzegorz Greczynski**: investigation, validation, methodology, writing – review and editing, formal analysis, data curation. **Per Eklund**: resources, project administration, writing – review and editing, conceptualization, investigation, funding acquisition, validation. **Ajay Soni**: conceptualization, investigation, funding acquisition, writing – review and editing, methodology, validation, supervision, resources, project administration, formal analysis, data curation.

## Conflicts of Interest

The authors declare no conflicts of interest.

## Supporting information




**Supporting File**: advs77576‐sup‐0001‐SuppMat.docx.

## Data Availability

The data that support the findings of this study are available from the corresponding author upon reasonable request.

## References

[1] Z. G. Yang , H. M. Xu , T.‐Y. Shuai , et al., “Recent Progress in the Synthesis of Transition Metal Nitride Catalysts and Their Applications in Electrocatalysis,” Nanoscale 15, no. 28 (2023): 11777–11800, 10.1039/D3NR01607B. 37404024

[2] H. Holleck , “Material Selection for Hard Coatings,” Journal of Vacuum Science & Technology A: Vacuum, Surfaces, and Films 4, no. 6 (1986): 2661–2669, 10.1116/1.573700.

[3] C. L. Chang , T.‐H. Chiou , P. H. Chen , W. C. Chen , C.‐T. Ho , and W.‐Y. Wu , “Characteristics of TiN/W_2_N Multilayers Prepared Using Magnetron Sputter Deposition with Dc and Pulsed Dc Powers,” Surface and Coatings Technology 303 (2016): 25–31, 10.1016/j.surfcoat.2016.03.086.

[4] H. D. Männling , D. S. Patil , K. Moto , M. Jilek , and S. Veprek , “Thermal Stability of Superhard Nanocomposite Coatings Consisting of Immiscible Nitrides,” Surface and Coatings Technology 146‐147 (2001): 263–267, 10.1016/S0257-8972(01)01474-8.

[5] L. Toth , Transition Metal Carbides and Nitrides, (Elsevier, 2014).

[6] B. Biswas , S. Chakraborty , A. Joseph , et al., “Secondary Phase Limited Metal‐insulator Phase Transition in Chromium Nitride Thin Films,” Acta Materialia 227 (2022): 117737, 10.1016/j.actamat.2022.117737.

[7] S. Gupta , A. Moatti , A. Bhaumik , R. Sachan , and J. Narayan , “Room‐temperature Ferromagnetism in Epitaxial Titanium Nitride Thin Films,” Acta Materialia 166 (2019): 221–230, 10.1016/j.actamat.2018.12.041.

[8] K. Ito , S. Honda , and T. Suemasu , “Transition Metal Nitrides and Their Mixed Crystals for Spintronics,” Nanotechnology 33, no. 6 (2021): 062001, 10.1088/1361-6528/ac2fe4. 34649229

[9] A. Kobayashi , T. Maeda , T. Akiyama , T. Kawamura , and Y. Honda , “Sputter Epitaxy of Transition Metal Nitrides: Advances in Superconductors, Semiconductors, and Ferroelectrics,” Physica Status Solidi A 222 (2025) 2400896, 10.1002/pssa.202400896.

[10] B. T. Matthias and J. K. Hulm , “A Search for New Superconducting Compounds,” Physical Review 87, no. 5 (1952): 799–806.

[11] R. Potjan , M. Wislicenus , O. Ostien , et al., “300 mm CMOS‐compatible Superconducting HfN and ZrN Thin Films for Quantum Applications,” Applied Physics Letters 123, no. 17 (2023): 172602, 10.1063/5.0176060.

[12] B. Pal , B. P. Joshi , H. Chakraborti , et al., “Experimental Evidence of a Very Thin Superconducting Layer in Epitaxial Indium Nitride,” Superconductor Science and Technology 32, no. 1 (2019): 015009, 10.1088/1361-6668/aaed8f.

[13] K. Takiguchi , Y. Krockenberger , Y. Taniyasu , and H. Yamamoto , “Berezinskii‐Kosterlitz‐Thouless Transition in Rhenium Nitride Films,” Physical Review B 110, no. 2 (2024): 024516, 10.1103/PhysRevB.110.024516.

[14] A. S. Ilin , A. O. Strugova , I. A. Cohn , et al., “Superconductivity in Thin Films of RuN,” Physical Review Materials 8, no. 7 (2024): 074801, 10.1103/PhysRevMaterials.8.074801.

[15] Y. Liu , Y. Liu , Z. Xu , et al., “From Weak to Strong‐coupling Superconductivity Tuned by Substrate in TiN Films,” Superconductor Science and Technology 37, no. 10 (2024): 105015, 10.1088/1361-6668/ad7642.

[16] S. Kalal , A. Tayal , S. Karmakar , R. Joshi , R. Rawat , and M. Gupta , “Electron–phonon Interactions and Superconductivity of β ‐Nb2N Thin Films,” Applied Physics Letters 122, no. 7 (2023): 072602, 10.1063/5.0142370.

[17] A. K. Verma , R. Gupta , S. Prakash , et al., “Structure and Superconductivity of Epitaxial and Polycrystalline VN Thin Films,” ACS Applied Electronic Materials 6, no. 7 (2024): 5029–5035, 10.1021/acsaelm.4c00540.

[18] A. Singh , D. Rawat , V. Hjort , et al., “Lattice Mismatch‐Driven in‐Plane Strain Engineering for Enhanced Upper Critical Fields in Mo_2_N Superconducting Thin Films,” Advanced Materials Interfaces 12 (2025): 00586.

[19] C. L. Bull , P. F. McMillan , E. Soignard , and K. Leinenweber , “Determination of the Crystal Structure of δ‐MoN by Neutron Diffraction,” Journal of Solid state chemistry 177, no. 4‐5 (2004): 1488–1492, 10.1016/j.jssc.2003.11.033.

[20] T. Takata , D. Lu , and K. Domen , “Synthesis of Structurally Defined Ta_3_N_5_ Particles by Flux‐assisted Nitridation,” Crystal growth & design 11, no. 1 (2011): 33–38, 10.1021/cg901025e.

[21] S. Wang , X. Yu , Z. Lin , et al., “Synthesis, Crystal Structure, and Elastic Properties of Novel Tungsten Nitrides,” Chemistry of Materials 24, no. 15 (2012): 3023–3028, 10.1021/cm301516w.

[22] A. Salamat , A. L. Hector , B. M. Gray , S. A. J. Kimber , P. Bouvier , and P. F. McMillan , “Synthesis of Tetragonal and Orthorhombic Polymorphs of Hf_3_N_4_ by High‐pressure Annealing of a Prestructured Nanocrystalline Precursor,” Journal of the American Chemical Society 135, no. 25 (2013): 9503–9511, 10.1021/ja403368b. 23721167 PMC3715886

[23] Y. Zhang , N. Haberkorn , F. Ronning , et al., “Epitaxial Superconducting δ‐MoN Films Grown by a Chemical Solution Method,” Journal of the American Chemical Society 133, no. 51 (2011): 20735–20737, 10.1021/ja208868k. 22126391

[24] N. Haberkorn , S. Bengio , S. Suárez , P. D. Pérez , M. Sirena , and J. Guimpel , “Effect of the Nitrogen‐argon Gas Mixtures on the Superconductivity Properties of Reactively Sputtered Molybdenum Nitride Thin Films,” Materials Letters 215 (2018): 15–18, 10.1016/j.matlet.2017.12.045.

[25] D. P. Dubal , N. R. Chodankar , and S. Qiao , “Tungsten Nitride Nanodots Embedded Phosphorous Modified Carbon Fabric as Flexible and Robust Electrode for Asymmetric Pseudocapacitor,” Small 15, no. 1 (2019): 1804104, 10.1002/smll.201804104. 30609283

[26] V. Chakrapani , J. Thangala , and M. K. Sunkara , “WO_3_ and W_2_N Nanowire Arrays for Photoelectrochemical Hydrogen Production,” International journal of hydrogen energy 34, no. 22 (2009): 9050–9059, 10.1016/j.ijhydene.2009.09.031.

[27] X. J. Ma and W. B. Zhang , “Tungsten Nitride for Capacitive Energy Storage,” ChemistrySelect 2, no. 28 (2017): 8726–8730, 10.1002/slct.201702007.

[28] X. Xu , F. Su , and Z. Li , “Tribological Properties of Nanostructured TiAlN/W_2_N Multilayer Coating Produced by PVD,” Wear 430–431 (2019): 67–75, 10.1016/j.wear.2019.04.021.

[29] M. Uekubo , T. Oku , K. Nii , et al., “WNx Diffusion Barriers between Si and Cu,” Thin Solid Films 286, no. 1‐2 (1996): 170–175, 10.1016/S0040-6090(96)08553-7.

[30] K. Balasubramanian , S. Khare , and D. Gall , “Vacancy‐induced Mechanical Stabilization of Cubic Tungsten Nitride,” Physical Review B 94, no. 17 (2016): 174111, 10.1103/PhysRevB.94.174111.

[31] J. Bekaert , C. Sevik , and M. V. Milošević , “First‐principles Exploration of Superconductivity in MXenes,” Nanoscale 12, no. 33 (2020): 17354–17361, 10.1039/D0NR03875J. 32789416

[32] V. Bagwe , R. Duhan , B. Chalke , J. Parmar , S. Basistha , and P. Raychaudhuri , “Origin of Superconductivity in Disordered Tungsten Thin Films,” Physical Review B 109, no. 10 (2024): 104519, 10.1103/PhysRevB.109.104519.

[33] J. A. Hofer , P. Pedrazzini , S. Bengio , and N. Haberkorn , “Superconductivity in Low Nitrogen Concentration Stabilized β‐tungsten Thin Films,” Materials Letters 360 (2024): 136007, 10.1016/j.matlet.2024.136007.

[34] J. A. Hofer and N. Haberkorn , “Superconductivity in Nanocrystalline Tungsten Thin Films Growth by Sputtering in a Nitrogen‐argon Mixture,” Thin Solid Films 685 (2019): 117–122, 10.1016/j.tsf.2019.06.014.

[35] A. Liang , I. Osmond , G. Krach , et al., “High‐Pressure Synthesis of Ultra‐Incompressible, Hard and Superconducting Tungsten Nitrides,” Advanced Functional Materials 34, no. 32 (2024): 2313819, 10.1002/adfm.202313819.

[36] Y. Asada and H. Nose , “Superconductivity of Niobium Films,” Journal of the Physical Society of Japan 26, no. 2 (1969): 347–354, 10.1143/JPSJ.26.347.

[37] V. M. Pan , V. P. Gorishnyak , E. M. Rudenko , et al., “Investigation of the Properties of Superconducting Niobium Nitride Films,” Cryogenics 23, no. 5 (1983): 258–262, 10.1016/0011-2275(83)90146-7.

[38] X. Zhou , C. Huang , J. Chen , et al., “Superconductivity in Hard Nitride cP6‐WN,” Physical Review Materials 10, no. 5 (2026): 054801, 10.1103/cjqb-59d6.

[39] M. Nazir , X. Yang , H. Tian , et al., “Investigation of Dimensionality in Superconducting NbN Thin Film Samples with Different Thicknesses and NbTiN Meander Nanowire Samples by Measuring the Upper Critical Field*,” Chinese Physics B 29, no. 8 (2020): 087401, 10.1088/1674-1056/ab9740.

[40] Y. Lee , J. Yun , C. Lee , M. Sirena , J. Kim , and N. Haberkorn , “Penetration Depth in Dirty Superconducting NbTiN Thin Films Grown at Room Temperature,” Applied Physics A 130, no. 7 (2024): 504, 10.1007/s00339-024-07650-0.

[41] D. Hazra , N. Tsavdaris , S. Jebari , et al., “Superconducting Properties of Very High Quality NbN Thin Films Grown by High Temperature Chemical Vapor Deposition,” Superconductor Science and Technology 29, no. 10 (2016): 105011, 10.1088/0953-2048/29/10/105011.

[42] M. Nikolo , J. Singleton , D. Solenov , J. Jiang , J. D. Weiss , and E. E. Hellstrom , “Upper Critical and Irreversibility Fields in Ba(Fe_0.92_Co_0.08_)_2_As_2_ and Ba(Fe_0.91_Co_0.09_)_2_as_2_ Pnictide Bulk Superconductors,” Journal of Superconductivity and Novel Magnetism 30, no. 3 (2017): 561–568.

[43] S. Khim , B. Lee , J. W. Kim , E. S. Choi , G. R. Stewart , and K. H. Kim , “Pauli‐limiting Effects in the Upper Critical Fields of a Clean LiFeAs Single Crystal,” Physical Review B 84, no. 10 (2011): 104502, 10.1103/PhysRevB.84.104502.

[44] A. Le Febvrier , L. Landälv , T. Liersch , D. Sandmark , P. Sandström , and P. Eklund , “An Upgraded Ultra‐high Vacuum Magnetron‐sputtering System for High‐versatility and Software‐controlled Deposition,” Vacuum 187 (2021): 110137.

[45] A. Le Febvrier , J. Jensen , and P. Eklund , “Wet‐cleaning of MgO(001): Modification of Surface Chemistry and Effects on Thin Film Growth Investigated by X‐ray Photoelectron Spectroscopy and Time‐of‐flight Secondary Ion Mass Spectroscopy,” Journal of Vacuum Science & Technology A 35, no. 2 (2017): 021407.

[46] G. Greczynski , D. Primetzhofer , J. Lu , and L. Hultman , “Core‐level Spectra and Binding Energies of Transition Metal Nitrides by Non‐destructive X‐ray Photoelectron Spectroscopy through Capping Layers,” Applied Surface Science 396 (2017): 347–358, 10.1016/j.apsusc.2016.10.152.

[47] International Organization for Standardization (ISO) 15472: 2010 Surface Chemical Analysis—X‐ray Photoelectron Spectrometers—calibration of Energy Scales, (International Organization for Standardization (ISO), 2010).

[48] C. Comparotto , P. Ström , O. Donzel‐Gargand , T. Kubart , and J. J. S. Scragg , “Synthesis of BaZrS_3_ Perovskite Thin Films at a Moderate Temperature on Conductive Substrates,” ACS Applied Energy Materials 5, no. 5 (2022): 6335–6343, 10.1021/acsaem.2c00704.

[49] K. Arstila , J. Julin , M. I. Laitinen , et al., “Potku—New Analysis Software for Heavy Ion Elastic Recoil Detection Analysis,” Nuclear Instruments and Methods in Physics Research Section B: Beam Interactions with Materials and Atoms 331 (2014): 34–41, 10.1016/j.nimb.2014.02.016.

[50] M. Mayer , SIMNRA User's Guide, (Max‐Planck‐Institut fur Plasmaphysik, 1997).

