# Supplementary material for: High Upper Critical Field and Type II Superconductivity in Epitaxial Cubic W2N Thin Films
Source: Adv Sci (Weinh). 2026 Sep 6:e77576. Online ahead of print. doi: 10.1002/advs.77576 (PMC13547794; doi:10.1002/advs.77576)
Supplement: Supplementary file 1 [file ADVS-9999-e77576-s001.docx]

**Supporting Information**

**High Upper Critical Field and Type Ⅱ Superconductivity in Epitaxial Cubic W_2_N Thin Films**

*Aditya Singh, Arnaud le Febvrier,* *Sanath Kumar Honnali, Abhisek Mishra,* *Grzegorz Greczynski, Subhankar Bedanta, Per Eklund^*^, and Ajay Soni^*^*

A. Singh, and A. Soni

School of Physical Sciences, Indian Institute of Technology Mandi,

Mandi 175005, Himachal Pradesh, India.

Email: [ajay@iitmandi.ac.in](mailto:ajay@iitmandi.ac.in)

A. L. Febvrier, S. K. Honnali and P. Eklund

Department of Chemistry - Ångström Laboratory;

Inorganic Chemistry, Uppsala University,

Uppsala, 75105, Sweden

Email: [per.eklund@kemi.uu.se](mailto:per.eklund@kemi.uu.se)

A. Mishra and S. Bedanta

Laboratory for Nanomagnetism and Magnetic Materials (LNMM), School of Physical Sciences, National Institute of Science Education and Research (NISER), An OCC of Homi Bhabha National Institute (HBNI), Jatni 752050

Odisha, India.

G. Greczynski and P. Eklund

Thin Film Physics Division,

Department of Physics, Chemistry, and Biology (IFM), Linköping University,

Linköping, SE-58183, Sweden.

The supporting information has the additional details of characterization and experiments complementing to the main text.

A. **X-Ray reflectivity of thin film (54 nm)**

**Figure S1:** XRR plot of the thin film (54 nm W_2_N/Al_2_O_3_) used grown in the optimized condition and used.

XRR measurements were performed to determine the thickness and deposition rate of W_2_N thin films. These measurements were carried out on reference epitaxial films (54 nm W_2_N/Al_2_O_3_) (Figure S1). This film was grown under identical sputtering conditions (identical gas flow, pressure, substrate temperature, and target power) as the film used for the transport properties characterization with a larger film thickness. The XRR data exhibited well-defined Kiessig fringes and were fitted giving a thickness of 54 nm. Since all films were deposited within the same calibrated process window, the reference films and the film used for the transport properties characterization are considered identical in terms of deposition rate. Using the extracted thickness and deposition time, the growth rate was determined and subsequently used to estimate the thickness of the superconducting W_2_N films studied in this manuscript, which were found to be approximately 270 nm.

B. **X-Ray diffraction of thin film and thick film**

**Figure S2** shows the XRD patterns of W_2_N thin film (54 nm) and thick film (270 nm) deposited on Al_2_O_3_ substrate. The films were deposited on the same day and under the same condition with a different duration of deposition. The diffraction peaks of both samples align well, confirming identical crystal structures, with reflections at 36.8° and 78.2° corresponding to cubic W_2_N, and peaks at 21.5° and 41.6° originating from the Al_2_O_3_ substrate.

**Figure S2:** XRD patterns of thin film with XPS composition of W_2_N_1.04_ and the thick film W_2_N/Al_2_O_3_.

C. **Pole Figure Analysis**

The in-plane orientation of the films is demonstrated by the pole figure analysis of the 200-reflections of W_2_N, observed at 2θ ~ 43.04° for W_2_N/Al_2_O_3_ and ~ 42.94° for W_2_N/MgO, as shown in Figure S3(a-b). The six poles of Al_2_O_3_ 11$\bar{2}$3, at Ψ = 61.3° and six poles of c-W_2_N 200, at Ψ = 56° in Figure S3(a) indicates epitaxial twin-domain growth, a common feature for cubic structures on both the substrates. In Figure S3 (b) three prominent poles are observed at Ψ = 55.5°, corresponding to MgO 200-reflections from the substrate alongside six poles from the W_2_N (200) planes, spaced every 60° in φ, with some overlapping the MgO poles. Both films epitaxially grow on c-plane sapphire and MgO (111) with no notable differences even in terms of twin domains which seems to be present on both substrates. The epitaxial relationship for both the films which are as follows: (111) W_2_N || (0001) Al_2_O_3_ (out-of-plane) and [11$\bar{2}$]W_2_N || [10$\bar{1}$0] Al_2_O_3_ (in-plane); and (111) W_2_N || (111) MgO (out-of-plane) and [110] W_2_N || [110] MgO (in plane).

**Figure S3:** Pole Figure XRD for (a)W_2_N/Al_2_O_3_ and (b) W_2_N/MgO.
